# In-Line Analysis of Diffusion Processes in Micro Channels by Long Distance Raman Photometric Measurement Technology—A Proof of Concept Study

**DOI:** 10.3390/mi12020116

**Published:** 2021-01-22

**Authors:** Julian Deuerling, Shaun Keck, Inasya Moelyadi, Jens-Uwe Repke, Matthias Rädle

**Affiliations:** 1 Center for Mass Spectrometry and Optical Spectroscopy, Mannheim University of Applied Sciences, 68163 Mannheim, Germany; s.keck@hs-mannheim.de (S.K.); i.moelyadi@hs-mannheim.de (I.M.); m.raedle@hs-mannheim.de (M.R.); 2 Process Dynamics and Operation, Technical University of Berlin, Fakultät 3 Prozesswissenschaften, 10623 Berlin, Germany; j.repke@tu-berlin.de

**Keywords:** process engineering, fluid-fluid extraction, Raman effect, photometry, microchannels, droplets, local concentration measurement, optical measurement

## Abstract

This work presents a novel method for the non-invasive, in-line monitoring of mixing processes in microchannels using the Raman photometric technique. The measuring set-up distinguishes itself from other works in this field by utilizing recent state-of-the-art customized photon multiplier (CPM) detectors, bypassing the use of a spectrometer. This addresses the limiting factor of integration times by achieving measuring rates of 10 ms. The method was validated using the ternary system of toluene–water–acetone. The optical measuring system consists of two functional units: the coaxial Raman probe optimized for excitation at a laser wavelength of 532 nm and the photometric detector centered around the CPMs. The spot size of the focused laser is a defining factor of the spatial resolution of the set-up. The depth of focus is measured at approx. 85 µm with a spot size of approx. 45 µm, while still maintaining a relatively high numerical aperture of 0.42, the latter of which is also critical for coaxial detection of inelastically scattered photons. The working distance in this set-up is 20 mm. The microchannel is a T-junction mixer with a square cross section of 500 by 500 µm, a hydraulic diameter of 500 µm and 70 mm channel length. The extraction of acetone from toluene into water is tracked at an initial concentration of 25% as a function of flow rate and accordingly residence time. The investigated flow rates ranged from 0.1 mL/min to 0.006 mL/min. The residence times from the T-junction to the measuring point varies from 1.5 to 25 s. At 0.006 mL/min a constant acetone concentration of approx. 12.6% was measured, indicating that the mixing process reached the equilibrium of the system at approx. 12.5%. For prototype benchmarking, comparative measurements were carried out with a commercially available Raman spectrometer (RXN1, Kaiser Optical Systems, Ann Arbor, MI, USA). Count rates of the spectrophotometer surpassed those of the spectrometer by at least one order of magnitude at identical target concentrations and optical power output. The experimental data demonstrate the suitability and potential of the new measuring system to detect locally and time-resolved concentration profiles in moving fluids while avoiding external influence.

## 1. Introduction

This article presents a new and fast method for the non-invasive, in-line quality control of local and time-dependent chemical composition and flow regime in microchannels by means of inelastic light scattering. All measurements were carried out at the Center for Mass Spectrometry and Optical Spectroscopy, an interfaculty institution of the University of Applied Sciences in Mannheim, Germany.

Temporally and spatially-resolved optical measurement techniques show great potential for improving insights into flow characteristics, mixing processes and therefore reaction monitoring, as well as process control in microchannels [1,2,3]. Established methods in the field of flow monitoring such as common image analysis provide 2-dimensional images, acquired by reflection or when using microchannels, transmission by means of specifically adapted and manufactured set-ups. This of course is predicated on both the bottom and lid of the channel being transparent [1].

There are various approaches to increasing image contrast, e.g., limiting the depth of field in conventional image analysis, since a narrowly defined detection plane results in a sharp image. Measuring a transmitted signal only gives information on mean concentration along the transmission axis. Although complex tomographic 3D-scanning instruments suppress this effect, increased acquisition times will be the consequence of capturing the necessarily high number of images [1].

Methods which combine microscopy and image analysis have made the spatial distribution of fluids and the temporal changes thereof accessible. Introducing contrasting agents into moving phases improves optical contrast. Although fluorescence markers require only low concentrations, they lack selectivity and possibly influence the fluids’ flow. Measuring auto-fluorescence is only rarely possible [1]. Consequently, the aforementioned techniques are unable to reliably detect local and temporal shifts in concentrations and are therefore insufficient for consistent tracking of mixtures, detecting possible occurrences of inhomogeneity, deviations from desired reactions and the formation of by-products. To fully address all of these needs it is paramount to have access to quantitative, molecule-specific information with a high time and local resolution without imposing external restraints on the flow regime.

Raman scattering is becoming more frequently used in microfluidics as a means of detecting target concentrations [4,5,6]. However, measuring techniques predominantly rely on spectrometers, as well as small focal lengths. Additionally, spatial resolution remains challenging. Consequently, the integration time for acquiring quantifiable spectra often lies in the range of seconds due to the low yield of the Raman effect. Fletcher et al. investigated the synthesis of ethyl acetate from ethanol and acetic acid inside a T-shaped channel using a Jobin Yvon Raman spectrometer and confocal optics, reporting acquisition times of 5 s per spectrum [6]. Rinke et al. monitored the hydrolysis of 2,2-dimethoxypropane to acetone and methanol in a T-shaped micromixer with a Leica microscope coupled to a Renishaw Raman spectrometer, with integration times ranging from 10 s to 60 s [5]. Gal-Or et al. presented the acquisition of Raman spectra of cyclohexane and ethyl acetate from 3D printed microfluidic devices by means of time-gated Raman spectrometry through picosecond-pulsed laser excitation, with measurement times of approximately 6 min per sample [4].

The presented measuring set-up distinguishes itself by utilizing recent state of the art customized photon multiplier (CPM) detectors and a long-distance non-contact probe, thereby addressing the issue of limiting integration times of spectrometers, while achieving the necessary spatial resolution for application in microchannel geometries. In this work, we will show that all criteria can be met using application-specific, preselected Stokes-Raman-Shifted information in a newly designed spectrophotometric Raman set-up.

## 2. Materials and Methods

The steadily increasing application of microstructured components in process engineering is a clear indicator of the growing importance of these elements in chemical, pharmaceutical and life sciences. Specific designs include heat exchangers, reactors, static mixers and others, and thus vary in size and shape [2,7]. The essential feature in all applications is the extremely high surface-to-volume ratio, stemming from small linear dimensions, which is the basis for the majority of advantages over conventional sized chemical process equipment [8]. This property improves selectivity and increases the yield of the chemical reaction, while also facilitating a more efficient heat transfer [7]. Furthermore, the divergent behavior of fluids in microscale dimensions in comparison to flow in macroscopic geometries must be considered. Due to dominating viscous effects, smaller dimensions result in low Reynolds numbers, which in turn impedes turbulent flow instabilities [9]. Accordingly, Newtonian fluids inside microstructured components such as microchannels will form laminar flow, along with its limiting influences on mass transfer and mixing [5].

**Figure 1:**
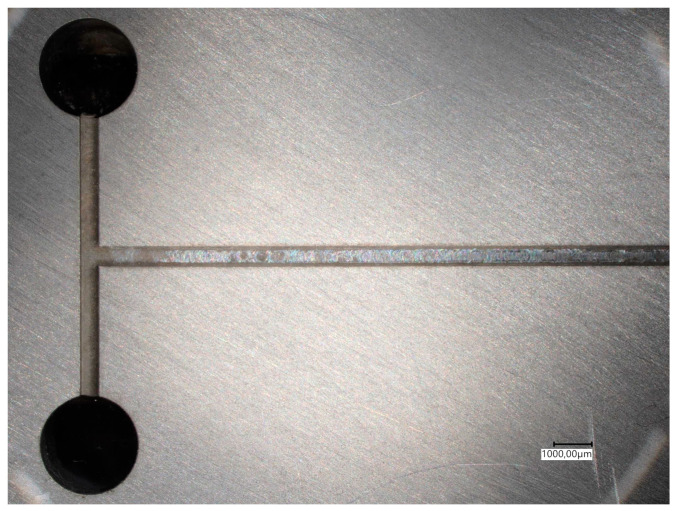
Top down view of the aluminium T-junction microchannel with two inlet and one output channels; square cross section of 500 × 500 µm, hydraulic diameter of 500 µm and 70 mm channel length (acquired with Keyence VHX-7000, Neu-Isenburg, Germany).

All mixing experiments were carried out in an aluminium T-junction microchannel (CN AW 2007 AlCu4PbMgMn). This alloy is specifically designed for milling while maintaining high tensile strength and is therefore easy to handle. Moreover, there are no interactions of this material with the fluid chemicals used in the experiments, making it ideally suited for the investigated fluid system. To reduce reflections and optimize wall flow the channel surfaces were homogenized by ball peening with spherical glass beads (40–70 µm; DIN 52 900). The microchannel had a square cross section with dimensions of 500 by 500 µm, a hydraulic diameter of 500 µm and 70 mm channel length, as shown in Figure 1.

The microchannel was covered with a transparent quartz lid for both visual and laser access to the flowing phases, as well as detecting inelastic scattered photons. Quartz glass causes a comparatively low background signal in regard to the common laboratory and industrial alternative borosilicate. To ensure leak tightness, the lid was fixed to the base body with a UV adhesive (Norland Optical Adhesive 61 LOT 415, Cranbury, NJ, USA). The adhesive was applied to the base body near the microchannel edges and was subsequently cured by UV light irradiation (Philips BLB F8T5, Amsterdam, The Netherlands). The reason for using this specific microchannel over conventional microfluidic devices was to verify the measurement system itself, before applying the system to more complex microchannels.

**Figure 2:**
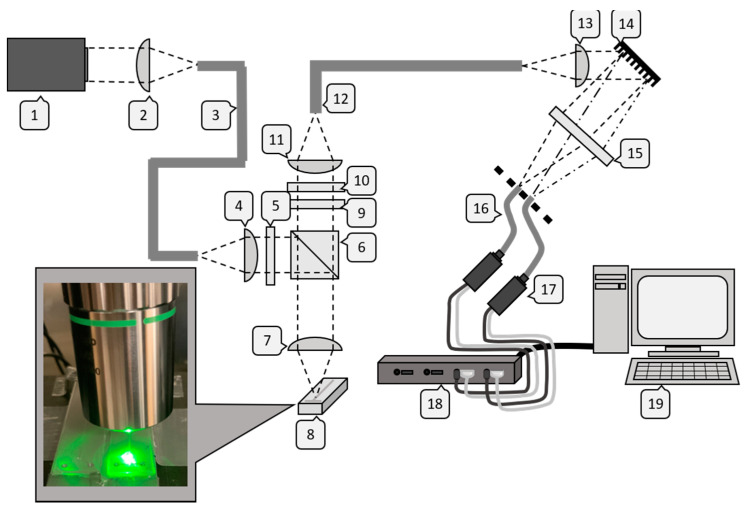
Optical set-up: laser stage #1–3, probe stage #4–7 and #9–12, microchannel #8, photometric detection stage #13–18, software #19.

The optical measuring system for monitoring of mixing consists of two functional units: the coaxial Raman probe with laser coupling and the spectrophotometric detector. The complete system is schematically depicted in Figure 2.

Both the excitation of Raman scattering and the collection of photons was realized by means of a specially designed non-invasive probe (#7 in Figure 2). Here, a fiber-coupled laser emitting at 532 nm (gem532, P_max_ = 500 mW, Coherent) in continuous wave mode was focused into the flowing liquid using a dichroic mirror and a long working distance (20 mm) microscope objective (Mitutoyo Apochromatic Objective, MY20X-804, Kawasaki, Japan) (#1–8 in Figure 2). The Raman effect was excited instantaneously and spherical scattering took place. The objective then collected inelastically scattered photons into a collimated beam, limited by its numerical aperture (NA = 0.42). The increased wavelength of Stokes-shifted Raman photons enabled passage through the dichroic mirror and blocking filter (#6,9,10 in Figure 2; AHF Analysetechnik). Photons were then coupled into a glass fiber (#12 in Figure 2). The probe was mounted vertically on a three-dimensional stage (Steinmeyer, MP130-50, Albstadt, Germany) with three linear axes for positioning during top down measuring. Detection of Stokes-shifted photons was accomplished using a novel spectrophotometric hybrid combining two key elements, a diffracting optic and a parallel alignment of glass fibers (#14, 15, 16 in Figure 2). Lenses (#2, 4, 12 and 13 in Figure 2) positioned before and after the glass fibers were used to focus or collimate the light. Inelastically scattered photons from inside the microchannel were relayed by glass fiber from the coaxial probe into a collimated beam and projected onto a holographic grating (grating constant 1800 mm^−1^, Thorlabs, Newton, USA). This type of grating characteristically has a low occurrence of periodic errors and low stray light; the latter is especially beneficial in measurements with a critical signal-to-noise ratio such as Raman spectroscopy. The emergent angle of incident photons from the grating surface was a function of the wavelength, enabling wavelength specific selection. The originally circular cross section of the beam was then focused in a linear shape onto the glass fibers. Fibers transferring relevant molecular information were then connected to customized photon multipliers (CM92N, Proxivision, Bensheim, Germany, #17 in Figure 2) and the information was then processed in additional hardware and software steps (#18, 19 in Figure 2). Optical power was measured at 70% of set power level at the probe head using a PS 19 thermopile sensor (Coherent, Santa Clara, CA, USA) in combination with a FieldMax II. The linearity of the excitation power was confirmed for the set-up with R^2^ = 0.9969, measuring the peak intensity of acetone at 1710 cm^−1^ Raman shift at various power settings.

**Figure 3:**
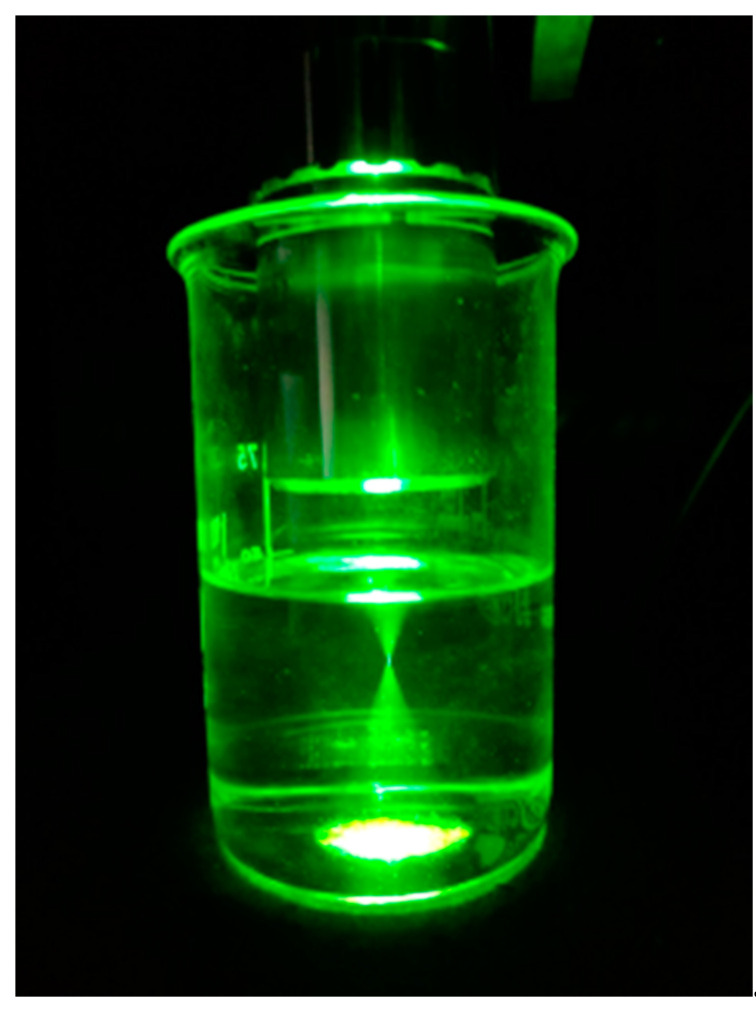
Laser-spot geometry (532 nm laser from coherent exiting probe head (#7 in Figure 2) in water).

The spot size and depth of focus of the laser focus are a defining factor of the spatial resolution of the set-up and these were measured using a Scanning-Split Optical Beam Profiler (BP209-VIS/M, Thorlabs). Several spot sizes were recorded at defined intervals along the optical axis. It should be noted that these measurements were done in free space. Since the refraction index of water and toluene is higher than air, the actual laser spot size will be smaller, thereby increasing the spatial resolution. The depth of focus was measured at approx. 85 µm with a spot size of approx. 45 µm (Figure 3). The working distance in this set-up was 20 mm and therefore suitable for application in microfluidics.

Customized photon multipliers were selected specifically for their applicability in low light detection. Their key features are extremely low background noise, low light level detection limits and high dynamic range and gain. In CPMs, sensitivity for large spectral ranges can be achieved by adapting the cathode material. With a signal amplification of 10^8^, they are among the most sensitive photodetectors. Furthermore, their low noise of < 10 cps (counts per second) is favorable when detecting low-yield Raman photons. The weak point of photon multipliers in general is their sensitivity to strong light influences (e.g., daylight, stray light, reflected light). The resulting high number of primary electrons leads to the destruction of the dynodes. In CPMs, such overexposure is prevented by an automatic quenching system that closes the entrance slit with a reaction time of 180 ns.

**Figure 4:**
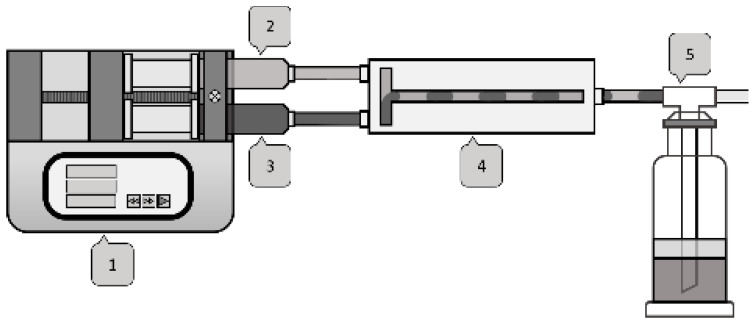
Fluid set-up: syringe pump #1, syringe with acetone and toluene #2, syringe with water #3, microchannel #4, waste container with mineral oil as diffusion barrier #5.

The substance system selected for the experimental series was toluene, acetone and water. As shown in Figure 4, the different fluids were pumped with a syringe pump into the microchannel at different flow rates.

**Figure 5:**
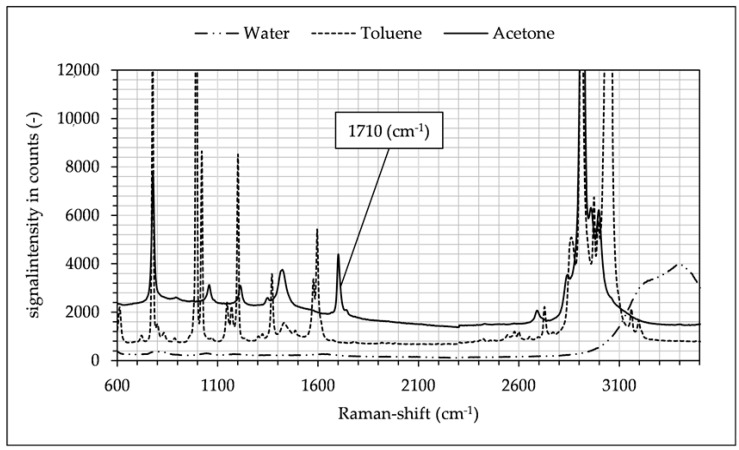
Raman spectra of toluene, acetone and water recorded with RXN1 spectrometer (Kaiser Optical Systems). The acetone peak at 1710 cm^−1^ used for concentration determination is framed. Spectra are offset by 500 and 1000 counts.

The pure substance Raman spectra were measured with a Raman spectrometer (RXN1, Kaiser Optical Systems, Ann Arbor, MI, USA) (see Figure 5).

**Figure 6:**
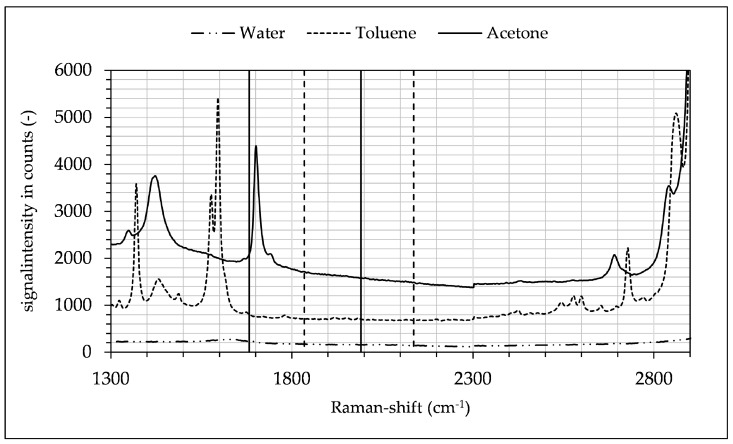
Section of Raman spectra of toluene, acetone and water. Spectral bandwidth of detection fibers are framed with vertical lines (bandwidth of maximum and minimum signal ~300 cm^−1^ each). Spectra are offset by 500 and 1000 counts.

This specific combination was chosen for several reasons. Firstly, the three-phase liquid–liquid extraction of this system is well documented in the literature [10,11,12,13]. Secondly, the spectral peak of acetone at 1710 cm^−1^ does not interfere with the peaks of toluene or water, as shown in Figure 5 and Figure 6. Moreover, the detection glass fibers had a spectral bandwidth of approx. 10 nm, which needed to be considered when aligning the central wavelengths of fibers. For these reasons the 1710 cm^−1^ acetone peak was suited for the detection despite its lower signal intensity, since in the investigated ternary system there was no overlap of any other peaks in the spectrum while using the spectrophotometric prototype. The band assignment (see Figure 5) for the substances used in these experiments was as follows in Table 1:

The spectral bandwidth of individual fibers translated to approx. 300 cm^−1^ in the current set-up. Peripheral fibers showed a slightly reduced signal intensity, stemming from a curvature in the focal plane. This was compensated for by using the signal intensity of the highest investigated concentration as a fixed reference for signal processing.

**Figure 7:**
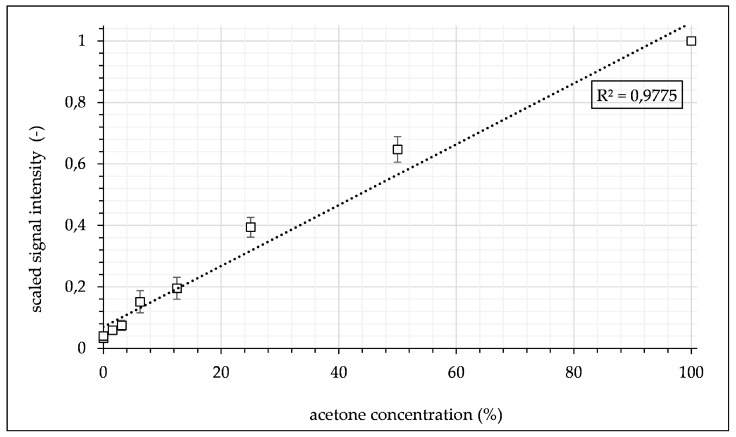
Calibration data of acetone signal intensity as a function of concentration measured during flow inside the microchannel. Data are scaled to maximum acetone intensity for comparability of different data sets.

Figure 6 shows a section of each Raman spectrum of the water–toluene–acetone system, with the solid and dotted vertical lines indicating the spectral ranges of the target and reference fibers. The overlap stems from a magnification of the terminal detection optics. In the investigated ternary system and the selected target range, this circumstance can be used to improve background signal correction, since the slope of the background can be better approximated as a photometric count rate due to the spectral proximity of the reference channel to the target channel. Using the prototype, binary mixtures were measured ranging from pure acetone to pure water with a minimum acetone concentration of 1.56% at an optical power output of 300 mW. Each mixture had a pre-selected, defined concentration and was continuously introduced into the microchannel. Every concentration consisted of 12000 individual data points. The signal was offset-corrected for empty conditions at approximately 5 counts. Calibration confirmed the linear behavior of the Raman effect as a function of concentration, with a Pearson coefficient of R² = 0.9775. The signal was then scaled to the maximum value of pure acetone, as depicted in the following Figure 7. Scaling facilitated the comparability of different experimental data sets, which varied between individual experiments while being self-consistent in each experiment.

For validation, raw data measured at different flow rates were treated accordingly and compared to this calibration curve.

## 3. Results

**Figure 8:**
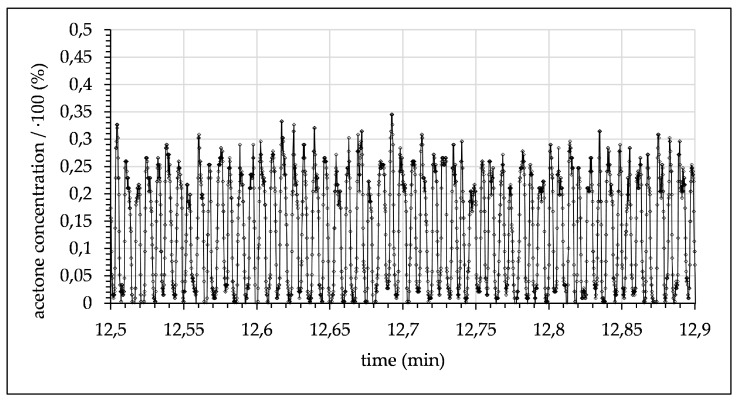
Measured acetone concentration in toluene droplets alternating with water droplets (flow rate 0.1 mL/min with 1.5 s time delay after contact of toluene and water).

In a first proof-of-principal experiment, acetone mixed with toluene at a ratio of one to three was introduced into the microchannel opposite pure water as the extracting solvent. A representative section of 24 s extracted from monitoring of these flow conditions (flow rate 0.1 mL/min) is depicted in Figure 8.

**Figure 9:**
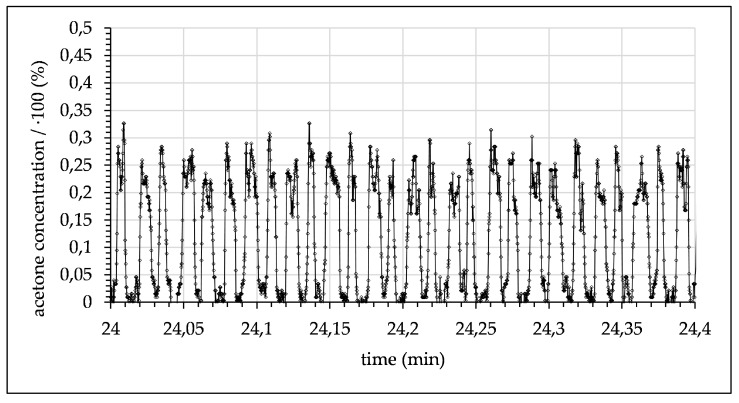
Measured acetone concentration in toluene droplets alternating with water droplets (0.06 mL/min with 2.5 s time delay after contact of toluene and water).

**Figure 10:**
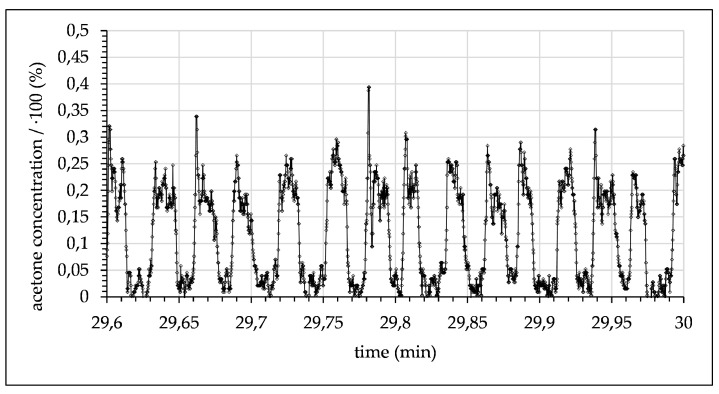
Measured acetone concentration in toluene droplets alternating with water droplets (0.04 mL/min with 3.75 s time delay after contact of toluene and water).

**Figure 11:**
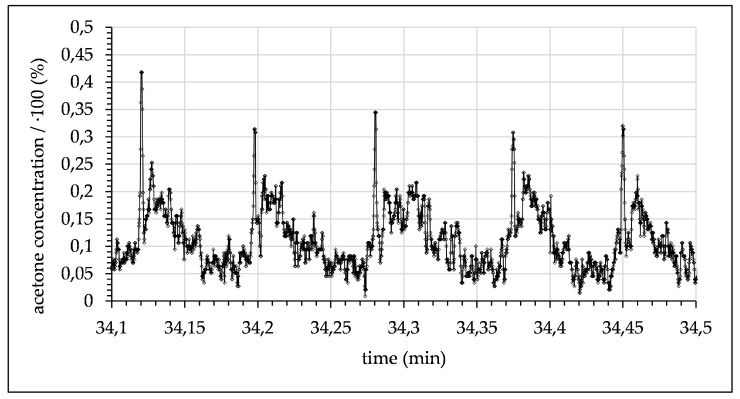
Measured acetone concentration in toluene droplets alternating with water phase (0.02 mL/min with a 7.5 s time delay after contact of toluene and water).

**Figure 12:**
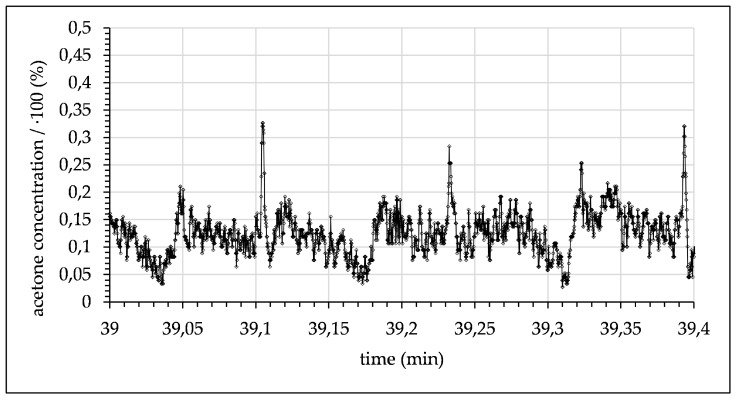
Measured acetone concentration in toluene droplets alternating with water phase (0.014 mL/min with a 10.71 s time delay after contact of toluene and water).

**Figure 13:**
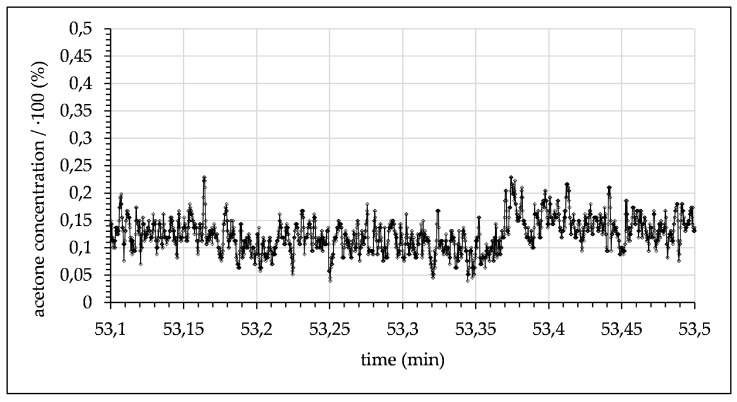
Measured acetone concentration in toluene droplets alternating with water phase (0.006 mL/min with a 25 s time delay after contact of toluene and water).

After mixing, droplets flowed through the microchannel until they passed the focal point where the concentration was measured. The signal for these experimental conditions periodically alternated between a maximum signal of ~24% (acetone concentration in toluene) and 0.9% (acetone concentration in water), both measured at a 10 mm channel length. The fixed measuring position and the varying flow rates resulted in differing time delays. The delays increased with the reduction of the flow rate and are given in the captions of Figure 8, Figure 9, Figure 10, Figure 11, Figure 12 and Figure 13. Although droplets of toluene and water were distinguishable, this indicates a negligible mass transfer of acetone from toluene to water at the given flow-rate. Consequently, to further investigate mass transfer, the flow rate was progressively decreased, thereby increasing the residence time. At 0.06 mL/min, this resulted in a maximum concentration of ~21.7% acetone in the toluene phase and ~2.15% in the water phase (see Figure 9).

At a flow rate of 0.04 mL/min (see Figure 10) there was slight decrease in the maximum signal to ~19.15% and an equal increase in the minimum signal to ~3.65%. The time periods of both the minimum and maximum signals were elongated, as was expected. Measurement artefacts appeared at some of the droplet boundaries.

The flow rate was further decreased to 0.02 mL/min. The local acetone concentration is shown in Figure 11. Here, a continuing decrease in the measured maximum concentration to ~16%, as well as an increase in the minimum concentration to ~8/.1% was displayed. In the region of the droplet boundary surface, a mixing concentration of ~12% became apparent. This mixing concentration seemed to be asymmetrical when comparing the front to the back of the droplet. In addition, the measurement artefacts were more pronounced.

A higher degree of extraction of acetone from toluene to water became visible at a flow rate of 0.014 mL/min (see Figure 12). The maximum and minimum concentration of acetone in both solvents further converged as it became increasingly difficult to distinguish the two phases.

Finally, at a flow rate of 0.006 mL/min (see Figure 13) the residence time was sufficiently long for half of the acetone inside toluene droplets to diffuse into the water phase, resulting in an acetone concentration of approx. 12.6%.

**Figure 14:**
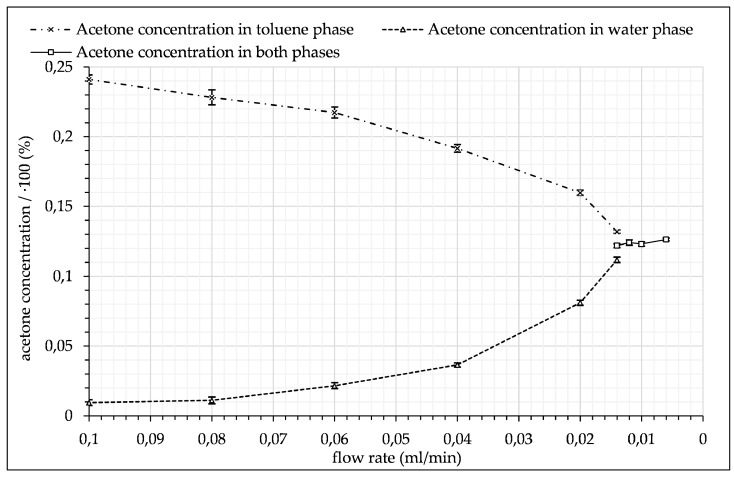
Acetone concentration in toluene droplets and water droplets as a function of the flow rate.

The experimental data are not shown in their entirety for all investigated flow rates, since there is no additional information to gain from the individual data sets. In the following section, the flow rates and corresponding concentration data are comparatively summarized in a single diagram for a comprehensive overview (see Discussion, Figure 14).

## 4. Discussion

The presented prototype for a Raman photometric top-down, long-distance monitoring set-up to detect mixing processes in microchannels was validated using the ternary system of toluene–water–acetone. The extraction of acetone from toluene at an initial concentration of 25% into water was studied as a function of flow rate and accordingly residence time was studied in a T-junction mixer. The investigated flow rates ranged from 0.1 mL/min to 0.006 mL/min. The corresponding residence times in the microchannel before reaching the measurement point ranged from 1.5 to 25 s. The chosen spectrophotometric detection was much faster, compared to the measurement of full spectra. Nevertheless, it isolated a single Raman peak specific to acetone and a reference section in close proximity to the spectral target range for signal processing. Toluene, as well as water, showed no Raman activity in the specified target range. A clear distinction of the immiscible phases was detectable with flow rates as low as 0.02 mL/min.

Regarding the measurement artefacts (seen in Figure 10, Figure 11 and Figure 12 as outliers), it is possible for the laser focal point to be refracted at the phase boundaries of the droplet, presumably enlarging the focal volume by entering the droplet not vertically, but at an angle. The artefacts only appear on one side of the droplet, as the boundary surface acts as a diffusing lens and then as a collecting lens when passing through the focal point. These phenomena can be ignored, as they do not represent the real acetone concentration and are therefore not included in the comparison data.

The experimental data demonstrate the principle’s feasibility and the potential of the new measuring system to detect locally and time-resolved concentration profiles in moving fluids without disturbing flow. The demonstrated results show the progressive transfer of acetone from the toluene phase into the water phase with increasing residence time. At 0.006 mL/min a constant acetone concentration of around 12% was measured, indicating that the mixing process had reached the equilibrium of the system of approx. 12.5%. Figure 14 shows the average acetone concentration in each phase as a function of flow rate until droplets were no longer distinguishable through acetone concentration alone. This trend shows the diffusion of acetone from the toluene droplet phase into the water phase until it reached an equilibrium state. All average concentration data are limited by the signal fluctuation, which is accounted for in the standard deviation. Furthermore, the standard deviation and the quantity of measurement points were used to calculate the standard error (see Figure 14).

When comparing the concave and convex droplet boundary surfaces (relative to flow direction), the data show a higher acetone concentration difference in “front” of the droplet (when comparing the two different phases) than in the “back”. This indicates different acetone mass transfer rates at the two points of the droplet, as seen in Figure 11. For benchmarking of the developed spectrophotometer, comparative measurements were performed with a commercially available Raman spectrometer (RXN1, Kaiser Optical Systems). Measured count rates of the prototype surpassed those of the spectrometer by at least one order of magnitude. Further improvements are under development.

This article focuses on the functional possibilities of the presented Raman photometric measuring system with a data rate of 100 measurements per second. To date, only the mean acetone concentration of droplets has been interpreted. Apparently, local distribution is consistently unequal inside of droplets. Further investigation of detailed transition processes will follow in a subsequent study. In this regard, next steps will include adapting the prototype to further optimize spectral resolution and integration time to elucidate local phenomena in two-phase systems.

## Figures and Tables

**Table 1:** Raman band assignment.

Water (cm^−1^)	Toluene (cm^−1^)	Acetone (cm^−1^)	Type of Vibration
	622, 787, 1005, 1091, 1211	787	C-H deformation
		1710	C-O stretching
		2921	C-H/C-H_2_ stretching
	3066		C-H/C-H_2_ stretching
>3200			O-H stretching

## Data Availability

The data presented in this study are available on request from the corresponding author.
